# A diagnostic dilemma: a case report

**DOI:** 10.1186/1757-1626-2-99

**Published:** 2009-01-29

**Authors:** David M Comer, J David M Edgar

**Affiliations:** 1 Level 8 Belfast City Hospital, Lisburn Road, Belfast, BT9 7AB, N Ireland, UK; 2 Immunology Day Centre, Royal Victoria Hospital, Grosvenor Road, Belfast, BT12 6AB, N Ireland, UK

## Abstract

**Background:**

A seventy nine year old lady presented with acute bilateral foot drop and paraesthesia of her lower limbs as a presenting feature of Wegener's Granulomatosis (WG).

**Case presentation:**

There was no evidence of pulmonary involvement and her renal function was normal. WG can masquerade as very diverse pathology. It is recognised that neuropathy can occur early and often in the absence of more classical pulmonary and renal findings, often resulting in a delay in diagnosis.

**Conclusion:**

Anti-neutrophil cytoplasmic antibody (ANCA) testing was particularly useful in this case permitting early diagnosis.

## Case presentation

A seventy nine year caucasian lady presented with bilateral foot-drop and lower limb dysaesthesia. This was of sudden onset. Left leg weakness and initial dysaesthesia of the left foot, progressed over 48 hours to involve her right leg. Apart from thoracic shingles nine months earlier and pulmonary tuberculosis in her youth, she had no significant past medical history. There were no upper or lower respiratory symptoms and she denied any joint pain, myalgia, sweats or haematuria. She was on no regular medication, was a non smoker, and consumed only minimal alcohol. Family history was unremarkable and she had no children.

On examination, there was a non-blanching erythematous reticular rash on the anterior aspect of both calves. She had bilateral foot-drop, with grade 0/5 power in all foot movements. Knee jerks were brisk, but ankle jerks were absent. She had bilaterally flexor plantar responses. There was loss of pin-prick sensation to mid calf bilaterally. Joint position sense and vibration sense were absent to below the knees. The remainder of the clinical examination was normal and she had a normal body mass index.

A myelogram and cerebrospinal fluid analysis was normal. Neurophysiology demonstrated normal responses in the arms but absent sensory and motor responses in both legs, in keeping with severe bilateral lumbosacral plexopathy or length dependent neuropathy. An MRI scan of her spine (to exclude a compressive lumbosacral polyradiculopathy) showed only mild age related cerebral atrophy and scattered degenerative changes. There was no evidence of spinal cord or nerve root compromise. Chest x-ray demonstrated evidence of old acid fast disease.

**Figure 1:**
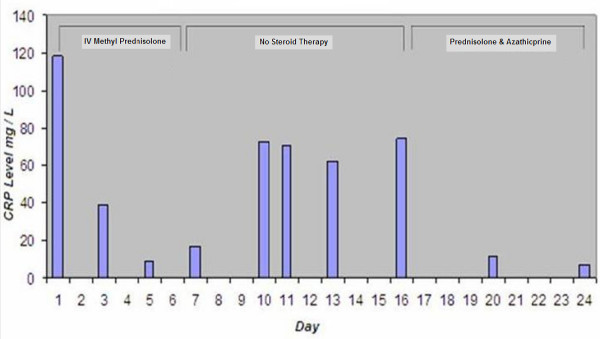
**Biochemical response to steroid and immunosuppressive therapy**.

Laboratory results including full blood picture, urea and electrolytes, fasting glucose, thyroid function tests, vitamin B12, folate levels and rheumatoid factor were all normal. Liver function tests were abnormal with an ALP of 515 U/l (35 – 120) and γ GT of 271 U/L (12 – 58). Hepatitis screening blood tests were normal and ultrasound scanning of her abdomen showed several gallstones with no evidence of biliary dilatation. She was treated empirically with intravenous methyl prednisolone (500 mg daily) for presumed myelitis for 5 days prior to the results of her MRI scan becoming available. Although she did not improve clinically with this regime, there was a striking fall in serum C reactive protein from an initial 118 mg/L to 8.8 mg/L over this period. 10 days after stopping methyl prednisolone, serum C reactive protein was 74.5 mg/L. Yet again this fell to 11.0 mg/L after 2 days resumption of prednisolone and azathioprine (Fig 1). ANCA results became available two days after admission. C-ANCA was positive at 1:320 dilution, PR3-ANCA was positive 42.1 U/ml (< 2 U/ml). This led to a working diagnosis of WG. Nasal septum mucosal biopsy revealed non-specific inflammation with no evidence of a vasculitic process. The patient declined a nerve biopsy. Together with a reducing course of steroids, she was commenced on azathioprine 100 mg daily, twelve days after completing the course of pulsed intravenous methylprednisolone.

12 months after the initiation of treatment she is clinically well, with almost complete resolution of bilateral foot-drop and no overt complications of treatment.

## Discussion

WG, a triad of granulomatous lesions involving the upper and lower airways, a systemic vasculitis and necrotising crescentic vasculitis involving the kidney, was first described in 1932 by Klinger et al [1]. Patients with WG usually present with symptoms and signs of upper airway or of lower airway disease. Less commonly patients present solely with symptoms of renal disease. The diagnosis of WG rests on the recognition of typical clinical features, supported wherever possible by confirmatory tissue histology. The advent of anti-neutrophil cytoplasmic antibody (ANCA) testing in the last 20 years has provided a valuable additional element in what can in certain cases be a very difficult diagnosis. Despite advances in our understanding of granuloma formation and improvements in treatment the pathogenesis of WG remains poorly understood. Treatment is based on combination immunosuppressive regimes to induce remission followed by lower dose maintenance therapy. Without treatment, the prognosis is poor, with a median survival time of four months [2]. With treatment, the chance of remission and long term survival is high with 7-year survival figures of 58% for those patients over the age of 60 and a corresponding value of 29% for those patients aged less than 60 [3].

This case was a diagnostic dilemma because an elderly female patient presented with peripheral neuropathy of uncertain origin. A common cause was not identified, but steroid therapy was commenced on an empirical basis. There was no obvious clinical responses in terms of weakness or sensation, however there was a dramatic response in terms of the detectable acute phase response. Of itself this suggested a steroid responsive inflammatory cause, however it was the detection of high titre C-ANCA, confirmed by positive PR3-ANCA that indicated the underlying diagnosis of WG. This provided justification for recommencement of immunosuppressive therapy, which continues.

Elderly people with WG present disproportionably with CNS involvement (4.5 fold more commonly) and less often with the classical symptoms [4]. Previous studies have shown that this cohort of patients characteristically present with symptoms and signs of pulmonary infiltrates and renal impairment rather than features of upper airway disease. This atypical presentation does not however imply a more indolent course and elderly patients require long term immunosuppressive treatment in the same way as their younger counterparts. Accurate confirmation or exclusion of the diagnosis of WG has enormous implications. An accurate and prompt diagnosis is potentially life saving, but the inappropriate use of immunosuppressive medication can have severe detrimental effects.

WG has been reported to present with diverse clinical signs including ulceration of the breast, pleural effusion, pituitary disease and as a Horners syndrome [5-8]. Although the detection of antineutrophil cytoplasmic antibody (ANCA) can be useful in these particular cases the results should be interpreted with caution. ANCA detection should be used as an adjunct for establishing a diagnosis of WG, but should not be used as a screening test in unselected cases. A positive test in the context of low pre-test probability may be a "false positive" result and alternative diagnosis should be considered. On the other hand, a high titre ANCA in the context of high clinical suspicion can enable the clinician to justify embarking on aggressive immunosuppressive medications. Clinical conditions recognised to cause "false positives" include the connective tissue diseases, malignancy and subclinical infections [9,10]. It is in the latter groups where inadvertent use of immunosuppressant medications could have devastating consequences. Audit data emphasises the caution required in interpretation of ANCA results by showing that the positive predictive value of ANCA of all types (including classical, perinuclear and intermediate staining patterns) was 27% in unselected requests. This figure however rose to 75% if the C-ANCA titre was particularly high (> 1:640) [9,10]. The further work of Rao et al confirmed and extended this observation that a positive ANCA in the context of a low pre-test probability of systemic vasculitis is likely to be a false positive result [11]. International guidelines for ANCA testing were developed (table 1) [12].

**Table 1:** Indications for ANCA testing [12]

Glomerulonephritis, especially rapidly progressive glomerulonephritis
Pulmonary haemhorrhage, especially pulmonary renal syndrome

Cutaneous vasculitis with systemic features

Multiple lung nodules

Chronic destructive disease of the upper airways

Long standing sinusitis or otitis

Subglottic tracheal stenosis

Mononeuritis multiplex or other peripheral neuropathy

Retro-orbital mass

In this case, ANCA testing was used appropriately, in line with these international consensus guidelines and proved useful in establishing a diagnosis. It is important to emphasise that ANCA testing should only be requested in these defined clinical situations and not be used as a screening test for vasculitis in patients with non-specific presentations [10].

## Consent

Written informed consent was obtained from the patient for publication of this case report and accompanying images. A copy of the written consent is available for review by the Editor-in-Chief of this journal.

## Competing interests

The authors declare that they have no competing interests.

## Authors' contributions

DMC was involved in preparing the manuscript. JDE is the consultant responsible for the patients care and edited the manuscript. All authors read and approved the final manuscript.
